# Innate Immune Memory in Invertebrate Metazoans: A Critical Appraisal

**DOI:** 10.3389/fimmu.2018.01915

**Published:** 2018-08-22

**Authors:** Daniela Melillo, Rita Marino, Paola Italiani, Diana Boraschi

**Affiliations:** ^1^Institute of Protein Biochemistry (IBP), National Research Council (CNR), Naples, Italy; ^2^Biology and Evolution of Marine Organisms (BEOM), Stazione Zoologica Anton Dohrn, Naples, Italy

**Keywords:** invertebrates, innate immunity, immunological memory, innate memory, immune priming

## Abstract

The ability of developing immunological memory, a characteristic feature of adaptive immunity, is clearly present also in innate immune responses. In fact, it is well known that plants and invertebrate metazoans, which only have an innate immune system, can mount a faster and more effective response upon re-exposure to a stimulus. Evidence of immune memory in invertebrates comes from studies in infection immunity, natural transplantation immunity, individual, and transgenerational immune priming. These studies strongly suggest that environment and lifestyle take part in the development of immunological memory. However, in several instances the formal correlation between the phenomenon of immune memory and molecular and functional immune parameters is still missing. In this review, we have critically examined the cellular and humoral aspects of the invertebrate immune memory responses. In particular, we have focused our analysis on studies that have addressed immune memory in the most restrictive meaning of the term, i.e., the response to a challenge of a quiescent immune system that has been primed in the past. These studies highlight the central role of an increase in the number of immune cells and of their epigenetic re-programming in the establishment of *sensu stricto* immune memory in invertebrates.

## Introduction

The phenomenon of innate memory in mammals is known since last century, based on observations that animals or cells in culture react differently to a stimulus, in terms of innate/inflammatory responses, if previously primed with the same or with a different agent (1–3). The phenomenon has been recently resumed with observations on the beneficial non-specific effects provided by vaccination (4). In experimental studies on severe combined immunodeficiency mice, lacking functional B and T lymphocytes, vaccination with Bacillus Calmette-Guérin (BCG) conferred cross-protection against non-mycobacterial diseases (5, 6). This immunological memory involved metabolic changes, leading to epigenetic re-programming of myeloid cells (7, 8). Upon *ex vivo* stimulation, monocytes from BCG-vaccinated individuals, as compared to cell from unvaccinated donors, showed an increased production of inflammatory cytokines and chemokines and increased release of reactive oxygen species for up to 3 months after vaccination (4). The “adaptive” behavior of monocytes/macrophages, after vaccination or infections, was evident as an increased phagocytic and microbicidal capacity upon a second challenge with the same or a different agent (9).

As an old biological process, innate memory evolved for protection of multicellular organisms, before the emergence of adaptive immune responses (10). In plants, which did not evolve mobile immune cells, localized pathogen attacks can elicit broad-spectrum immunity to reinfection throughout the whole body, an immune memory phenomenon known as Systemic Acquired Resistance (SAR). SAR can last from few days to the full lifespan, and can be inherited (11). The molecular mechanisms and biochemical mediators of SAR are well known (12), with epigenetic re-programming of host defense playing a central role (11–13). In the last decades, a plethora of immunological studies on invertebrate metazoans suggest that their innate immune system also displays memory traits [reviewed in (10, 14, 15)]. The recent growing efforts in elucidating innate memory mechanisms in vertebrates would greatly benefit in using non-vertebrate models as benchmark of innate memory mechanisms, as in these animals the confounding element of adaptive immunity is not present. To this end, here we will critically examine the evidence and mechanisms of immune memory in invertebrates, in order to provide a solid and reliable picture of the phenomenon. In this view, we would like to stress that we will focus on studies showing the resolution of the priming infection before a second exposure (“extinction”), i.e., those describing immune memory in the strict sense of the term. Thus, we will not consider, although excellent, the wealth of data addressing immune memory in a larger meaning, i.e., the modulation of subsequent responses in animals already primed or activated, as these are describing immune reactivity more in general, and not only the memory phenomena.

## Environment and the immune system

Invertebrates represent 97% of animal diversity and can be found practically in any environment. This wide diffusion implies that each species should be able to adapt and survive in its environment by only relying on the defense mechanisms of innate immunity. The mechanisms of immune memory are therefore central to the invertebrate capacity of surviving in diverse environments.

Like in plants, in which priming can be induced both by biotic and abiotic environmental stimuli (16, 17), metazoan immunity is responsive to environmental cues in terms of learning experience that allows an individual to adjust its functional immune phenotype in response to subsequent stimuli (18, 19). Thus, the study of primitive metazoans could provide not only information on their global defense reactivity but also hints on the correlation between their lifestyle (e.g., sessile, colonial, social, etc.) and the prevailing type of immune reactions (e.g., phagocytosis, cytotoxicity, etc.).

A good example comes from mollusc classes. The availability of molluscan genomes highlights a substantial diversity in the immune features in gastropods and bivalves, the two major molluscan classes with adaptation to different habitats and with distinct lifestyles. In bivalves, lectin-like gene families undergo a greater expansion and diversification both in sequence and in the carbohydrate recognition domains. Conversely, in gastropods, the sequence diversity is limited, and it seems to be compensated by somatic diversification, as a great number of somatic mutations have been reported (20). At the level of effector mechanisms, it has been suggested that the rate of phagocytosis in gastropods depends on the concentration of plasma lectins, as opposed to bivalves, where opsonization is triggered by membrane-bound lectins (21, 22). The development of different phagocytosis-inducing strategies (agglutination mediated by membrane lectins vs. opsonization mediated by soluble lectins) could be an adaptation to different environments (terrestrial vs. freshwater vs. marine) and to the environment-associated microbiota and pathobiota (20). Redundancy, compensation and the consequent possibility of using different mechanisms for reaching the same result is in fact a typical characteristic of immunity [see for instance (23)]. Likewise, we can hypothesize that the re-programming at the basis of immune memory will selectively involve the defensive mechanisms that are preferentially used by each invertebrate class.

Diversity-generating mechanisms have been proposed as a possible way used by invertebrates for establishing specificity of response and immune memory. In the snail *Biomphalaria glabrata*, 13 different families of the immune-related Fibrinogen-Related Proteins (FREPs) are present, and 314 different sequences were observed for one of the FREP genes (FREP3) (24). The alternative splicing of the Down syndrome cell adhesion molecule (Dscam) was proposed as a potential mechanism for generating long-lasting immune responses in insects and in crustaceans. The hypervariability in Dscam splice isoforms is most likely involved in parasite recognition, and its involvement in immune memory is an important possibility, although formal proof is still needed (25). In the sea urchin *Strongylocentrotus purpuratus* it was possible to identify, based on homology, 222 TLR genes, and 203 NOD/NLRP-like genes (26). Whether expression of these genes changes upon repeated infection/stress is however still largely unknown. One of the few examples of memory-associated diversification, again in sea urchins, regards the pathogen-recognizing soluble proteins of the 185/333 family, a rapidly diversifying gene cluster, whose expression is induced by diverse types of biotic and abiotic stress (27). Upon repeated infections, the 185/333 proteins change in size and charge, as a consequence of mRNA editing and post-translational processing. Most likely, these changes are aimed at improving the recognition of and defense against pathogens (28).

Insects exploit two different RNA-based pathways against viral infection, short interfering RNAs (siRNAs) and PIWI-interacting RNAs (piRNAs) (29). In *Drosophila melanogaster*, production of siRNAs occurs in haemocytes following uptake of viral RNA. When transferred to naïve animals, these anti-viral siRNAs confer passive protection against virus infection, similarly to passive transfer of antibodies in mammals (30). Transcription of piRNAs from endogenous viral elements, integrated in the host genome after viral infection, has been described in *Aedes aegypti*, a phenomenon that implies the heritability of specific anti-viral protective effectors (31). Both mechanisms, i.e., passive and active protective interfering RNAs, are specific for the primary viral infection and can be considered as adaptive immune memory mechanisms.

An important notion comes from studies of immunity in the context of the natural environment of the organisms, which has been critical for the recognition of the complex system of immunological and non-immunological host defense strategies (32). Fundamental to this approach (ecological immunology) is the awareness that immunity is energetically costly to organisms in terms of using and maintaining an immune system. Thus, protective mechanisms also take advantage of traits directly tied to host fitness, as for instance hygienic behavior (33), self-medication (34), social immunity (35), fecundity compensation (36), symbiont protection (37, 38), etc. Non-immunological defense can stimulate immune-based defense functions, and vice versa. In the nematode *Caenorhabditis elegans*, the interaction with pathogenic bacteria generates a conditioned behavior, which causes avoidance of bacteria upon a subsequent exposure, based on different mechanisms of olfactory sensing (aversive olfactory learning through increase in serotonin, and food-leaving behavior involving the Toll-like receptor TOL-1) (39, 40). It is hypothesized that immune signals generated during the first encounter with pathogens may contribute to this olfactory/neurological imprinting (39).

Based on all the above considerations, it is important to stress the importance of the experimental laboratory settings used in the studies on invertebrates. In fact, in order to obtain reliable results, it is important that the laboratory conditions reproduce the environmental conditions under which hosts and microorganisms/stressors interact in nature. For example, studies might fail to measure an immune response in some organisms in a lab setting only because in the natural environment immune responses also depend on non-immunological defensive mechanisms and on the complex environment in which the reaction occurs.

### Natural transplantation immunity

A phenomenon strictly correlated to the environmental living conditions, the so-called natural transplantation immunity, has been considered as a way of assessing immune memory responses.

This phenomenon is typical of colonial sessile marine invertebrates (*Porifera, Cnidaria, Urochordata*), hypothetically as a strategy to prevent competition for substrate. Natural transplantation immunity, or allogeneic cytotoxicity, would allow individuals in sessile colonies to recognize individuals with a different genotype, and to react against them.

In *Porifera*, the phenomenon of fusion between tissues of genotypically different individuals has not been observed in the wild, for instance in the case of colonies of *Callyspongia diffusa*, not even when different colonies grow in close proximity or contact. The phenomenon has been described only in the lab in allogeneic tissue transplantation experiments, in which the rejection of the second graft of an incompatible tissue is faster than the first time, the effector mechanism consisting in the faster release of cytotoxic proteins (41).

On the other hand, the colonial urochordate *Botrillus schlosseri* undergoes a natural “transplantation” reaction upon contact with a different individual. The interaction can have two outcomes, depending on the genetic compatibility of the interacting colonies (42). Fusion can occur, with a vascular reorganization and formation of new blood vessels that allow the fusing colonies to share the blood supply. On the other hand, an inflammatory rejection reaction can take place, causing the detachment of the two individuals. The rejection response begins with the migration of a particular type of haemocytes, the morula cells, into the tips of the interacting colonies, where they discharge the content of their vacuoles and initiate an inflammatory reaction that includes the formation of phenoloxidase (PO)-dependent melanin scars, the so- called “points of rejection” (42). Both outcomes are controlled by a single fusibility/histocompatibility (*Fu/HC*) locus with multiple co-dominantly expressed alleles (42). In the case of fusion, it is interesting to see that one genotype dominates on the other, as gametes belong to only one of the fusing partners (43). Thus, this process is not a true fusion, and is therefore called “germ cell parasitism”. The ability to parasitize or to be parasitized is heritable (44).

In the cnidarian genus *Hydractinia*, colonies growing in contact undergo an allorecognition response that often results in rejection and only rarely in fusion (45). Contact between different colonies induces a recruitment of nematocytes (cnidarian defensive cells) in the contact areas of both colonies. In the case the two colonies are not compatible, nematocytes discharge their nematocysts, harpoon-like organelles that cause damage to the tissues of the adjacent colony and *de facto* disconnect the two colonies (46). Conversely, in the case of compatible colonies, nematocytes leave the contact area, ectodermal cells of the two colonies adhere to each other, and functional gastrovascular continuity is established (46). Fusion is governed by two highly polymorphic loci, *alr1*, and *alr2*, each with multiple alleles. Colonies can fuse when sharing at least one allele at both loci (47).

In summary, in species that naturally grow in contact, rejection of incompatible colonies can occur, with necrotic damage to the allogeneic tissues (42, 46). However, from an accurate analysis of the data, including those mentioned above, there is no evidence of activities or mechanisms that can be ascribed to immune memory *sensu stricto* (10).

## Evaluation of immune memory in invertebrates

**Figure 1:**
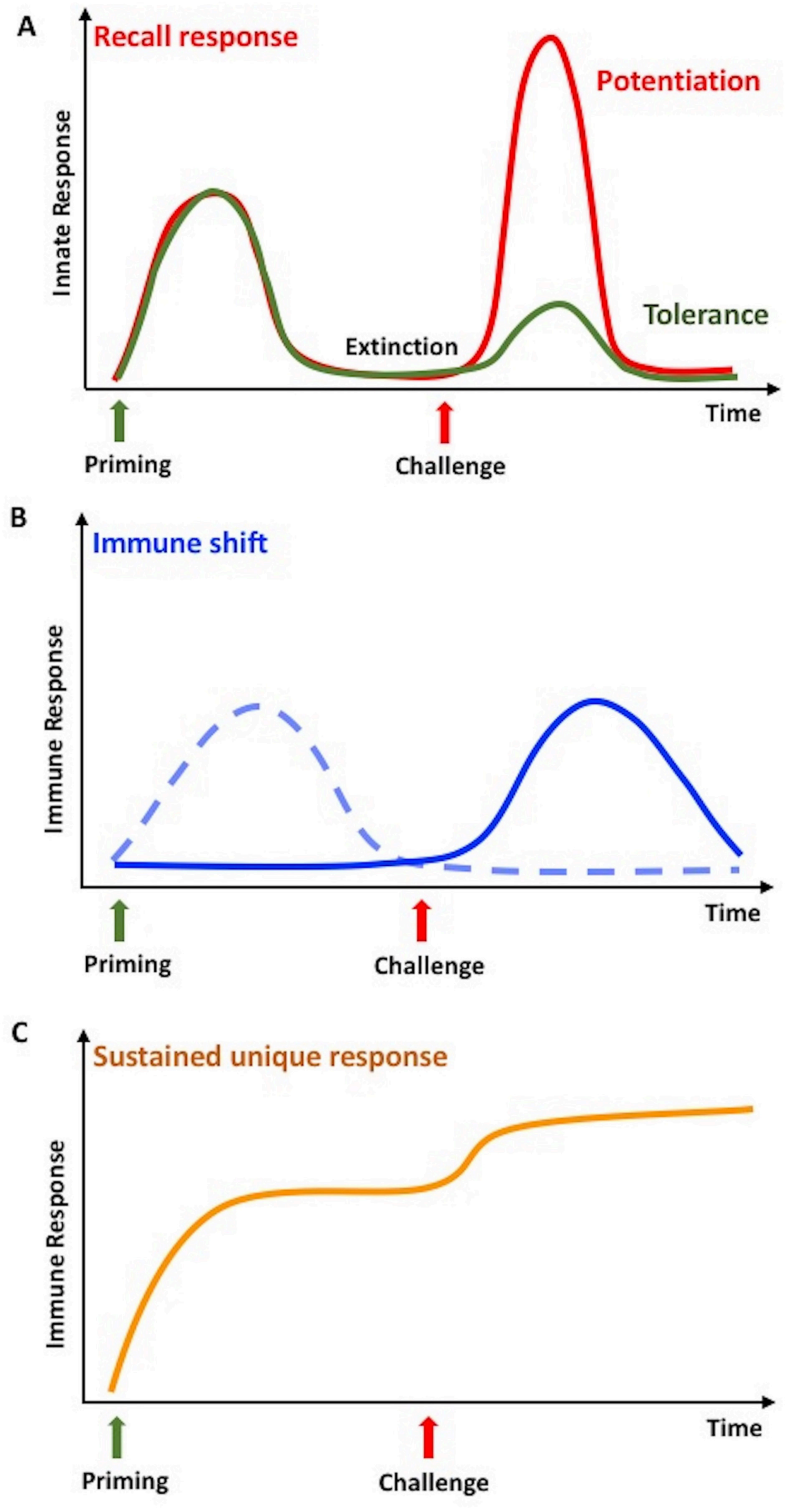
Innate memory in invertebrates and vertebrates. **(A)** Infections or stressors can prime the innate immune system so that, after a phase of extinction of the response, it will respond more potently to a subsequent challenge with the same or a different stimulus. In invertebrates, this is defined as a recall response, while in vertebrates it is called potentiation or trained immunity (red line). Only in vertebrates, the secondary response can be less intense than the first one, a phenomenon known as tolerance (green line). **(B)** In invertebrates, a second challenge in primed animals can lead to an immune shift, i.e., the shift from a type of response (dotted line) to a different, more efficient one (solid line). **(C)** In invertebrates, priming could result in a medium- or long-term immune activation state, which can further increase upon challenge. This is identified as sustained or unique response. Adapted from Coustau et al. (48) and Pradeu and Du Pasquier (15).

In vertebrates, innate immune memory is a stimulus-induced re-programming of innate immune functions, resulting either in decreased reactivity (tolerance) or enhanced responsiveness (potentiation) to a subsequent challenge (Figure 1A). In both cases, establishment of innate immune memory has the main goal of better defending and preserving the integrity of the organism. In fact, enhanced reactivity could eliminate subsequent infections more efficiently, whereas tolerance could limit the tissue-destructive *sequelae* of excessive or persistent immune activation. This may however translate, in the long term, also into decreased resistance to infections or increased side effects (49, 50). The analytical tools for evaluating innate immune memory are increased resistance to infections *in vivo* in the whole animal/individual, or a simple evaluation of cytokine production, gene expression, or up/down-regulation of surface markers in monocytes or macrophages challenged in culture after a priming *in vivo* or *in vitro* (7, 51). It is important to note that the phenomena of potentiation and tolerance are those that we can observe at the level of the entire organism, while at the level of single cells/cell populations we can observe a re-programming of their activation in directions that cannot be immediately ascribed to overall potentiation or overall tolerance (52). It is therefore important, when addressing innate immune memory both in vertebrates and invertebrates, to specify whether we are considering the overall effect at the organism's level, or the molecular mechanisms underlying induction of memory at the cellular level.

Immune memory has been recently defined as a multidimensional phenomenon, in which different mechanisms and dimensions contribute to the response, and in which the dimension of “extinction” of response after the first stimulation defines the true re-activation and memory response (15). In invertebrates, the term immune memory is used for describing at least three different phenomena, of which probably only the first two are, strictly speaking, true memory phenomena (Figure 1). The three phenomena are described below.

1. Immune memory generates a **“recall” response** that, after a phase of extinction of response, is faster and more powerful than the first time, upon exposure to either related or unrelated microbial stimuli (53–56). This phenomenon is a *bona fide* memory response that we can observe also in vertebrates.
2. Some studies have described memory as a **shift from one type of response to a different one**, more efficient in clearing the foreign agents (56). This is as well a *bona fide* memory phenomenon.
3. A process of **acquired resistance or sustained unique response** is also defined as immune memory, and consists of a long-lasting up-regulation of the defense activity (15, 57). The phenomenon however does not allow distinguishing whether, within this persistent activation, is active a phenomenon of memory *sensu stricto*, i.e., the re-programming of immune cell reactivity.

With the availability of invertebrate genome sequences, many genes and molecules have been identified in early invertebrates, based on sequence homology with vertebrate immune-related genes. However, studies on their functions are limited to a few molecules in a few species, in particular *Drosophila* for Pattern Recognition Receptors (PRRs) (58, 59), and *Ciona intestinalis* for complement (60, 61). Consequently, the functional and valid identification of the molecular pathways underlying invertebrate immune responses, including immune memory, is still at its infancy. This is why, in invertebrates, we can evaluate the generation of immune memory by using a limited number of parameters/functional phenotypes (62), as briefly described below.

- Percentage of survival upon repeated infections.
  The most used way of assessing immune memory is the evaluation of survival to an infectious challenge in previously primed animals vs. naïve controls.
- Efficiency of parasite/pathogen clearance.
  Pathogen clearance is usually measured in animals infected with a non-lethal pathogen dose. At different times after infection the number of surviving microorganisms is assessed, usually by plating tissue homogenates from infected animals on appropriate growth media and counting colonies.
- Heritability of enhanced resistance.
  In some invertebrate metazoans, immune memory can be transmitted to the progeny (transgenerational immune priming). Resistance to infections in the unprimed progeny of primed animals is used for assessing the heritability of immune memory.
- Index and rate of phagocytosis.
  Phagocytosis, the most used immunological parameter in invertebrates, is assessed as phagocytic index (number of phagocytosed particles/bacteria per phagocytic cell) and as rate of phagocytosis (percentage of phagocytosing cells within the total phagocyte population, although several authors calculate it on the total haemocyte number). An increased phagocytic index describes the re-programming of cell functions, whereas an increased phagocytic rate implies an increase in the number of phagocytic cells (not in their efficiency).
- Resistance induced by cell-free haemolymph transfer.
  In several studies the transfer of cell-free haemolymph from primed to naïve individuals is used for demonstrating the priming-induced generation of protective immune soluble factors that can cause protection in unprimed individuals. It is however quite clear that this is not a way for measuring immune memory, since this is a passive transfer of bioactive molecules, acting either directly on the infectious agent (e.g., antimicrobial peptides) or by promoting immune activation (e.g., factors inducing phagocyte differentiation).
- Resistance induced by tissue transplantation.
  Tissue transplantation is used in *Planaria* for transferring stem-like cells (which, upon transplant take, differentiate into immune cells) from primed animals to naïve recipients. Such transfer results in the accelerated activation of anti-infective mechanisms upon bacterial challenge, a typical recall response.
- Increased expression of immune-related genes.
  The increased resistance to infection upon repeated exposure to stimuli is often correlated to a variation in the expression of some immune-related genes, even though in many cases the real involvement of such genes in a defensive activity is still not formally proven.
- Candidate-free transcriptomic analysis.
  The transcriptional analysis of changes induced in immunocompetent tissues by priming/activation (63), and the new gene expression repertoire evoked by challenge in primed individuals (64) provide an excellent molecular basis to the phenomenon of immune memory, which will need functional validation of identified genes and pathways.
- Accelerated rejection of allo- and xeno-transplants.
  Rejection of allo- and xeno-transplants in earthworms has been widely and successfully used for assessing immune responses and memory. Allograft rejection in *Lumbricus terrestris* is faster in animals already transplanted vs. controls, but this priming is short-lived, fading after 10 days from the first transplant (65). Transfer of coelomocytes from *L. terrestris* previously xeno-transplanted with tissue from *Eisenia foetida* could achieve faster rejection of the xeno-transplant in unprimed recipients (66). The authors of these studies hypothesize that the phenomenon is likely due to the persistence of transplant-activated defensive cells (a sustained response).

From the above list, it is evident that the evaluation of immune memory in invertebrates needs more information and tools, starting from the availability of better direct information on the function (and in many cases also the protein product) of the homology-identified immune-related genes, and ending with a larger number of functional assays. We also wish to repeat a comment made previously, i.e., that the lab conditions in which experiments are run, if failing to reproduce the natural environment of the organism, could deeply affect the results.

The invertebrate organisms that are best studied for immune memory belong to coelomate metazoans, in which the circulating haemocytes are the major immune effector cells. As these cells are also those involved in the development of immune memory, we will briefly describe them.

## Haemocytes

**Figure 2:**
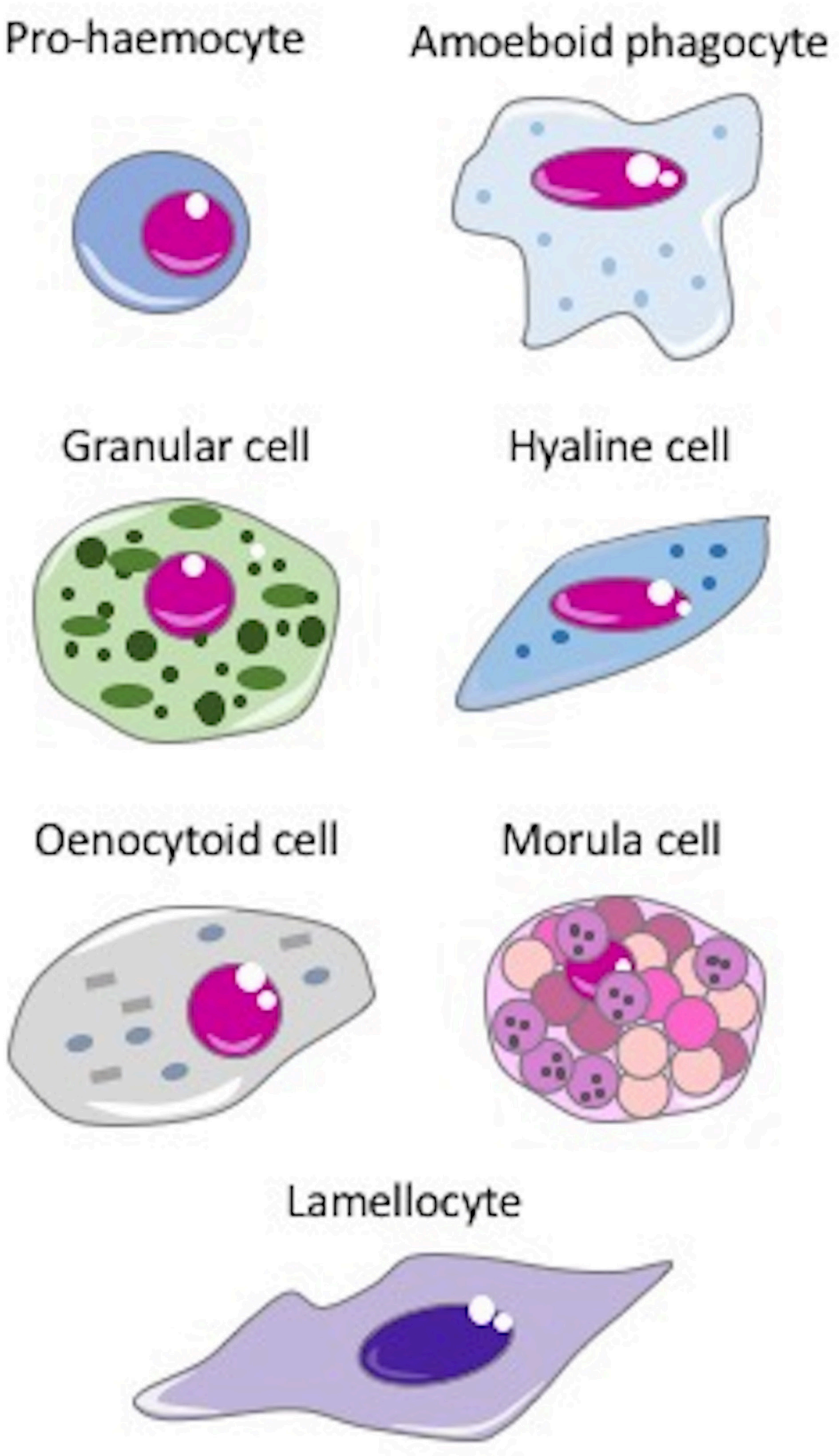
The main invertebrate haemocytes involved in immune response. **Pro-haemocyte**. Immature cell identified as pro-haemocyte or lymphocyte-like cell. These cells, present in ascidians, crustaceans, insects and probably in the haemopoietic tissues of other invertebrates, are able to differentiate in mature haemocytes. The undifferentiated pro-haemocyte is small, with a big nucleus containing a large amount of heterochromatin and a prominent nucleolus. **Amoeboid phagocytes** are motile vacuolated cells present in annelids, insects, echinoderms and ascidians. Depending on the species, amoeboid phagocytes are involved in phagocytosis, migration, wound repair, non-self-recognition, transplant reaction, cytotoxicity, encapsulation, endocytosis, and enzymatic digestion of engulfed material. **Granular cells** are mature cells found in ascidians, crustaceans, insects and bivalves. They are able to synthesize a number of cytotoxic and defense factors and store them in granules. Degranulation occurs upon challenge with stressors. **Hyaline cells** are vacuolated or non-vacuolated cells, abundant mostly in ascidians, crustaceans, and bivalve molluscs. They are mainly involved in phagocytosis. In ascidians, hyaline cells rapidly clump together *in vitro*. **Oenocytoid cell**. These cells are widely present in insect species. They are large cells with a low nuclear-cytoplasmic ratio, which show phenoloxidase activity in the cytoplasm. This suggests that oenocytoid cells could play a role in the melanization process. **Spherule or morula cell**. These haemocytes, present in some cnidarians, annelids, insects, echinoderms, and ascidians, are berry-shaped cells, sometimes pigmented, with highly refractive cytoplasmic inclusions. They are actively involved in encapsulation and synthesize, transport and release various defensive factors during infections, including antimicrobial proteins, cytotoxic factors, and opsonins. **Lamellocyte**. These flat cells with adhesive properties are present in insects, in particular in *Diptera*. Lamellocytes appear in the lymph glands and haemolymph during larval development and differentiate in response to parasite infection. They are active in neutralizing and encapsulating materials recognized as “non-self,” too large to be phagocytosed.

Haemocytes are present in the vascular lumen and the coelomic cavity of all coelomate animals. In invertebrates, many types of haemocytes have been described, with increasing diversity and specialization depending on body size and anatomical complexity (67). Some of the main types of haemocytes are depicted in the Figure 2. Haemocytes are generally endowed with defensive capacities, namely phagocytic, encapsulating, and microbicidal activities that contribute to both cellular and humoral responses to insults. The most common defensive haemocyte types are phagocytic and granular cells.

Granular haemocytes are cytotoxic cells that synthesize and store bioactive molecules within granules. Granules are discharged (degranulation) when cells are exposed to endotoxin, foreign materials or foreign cells (68, 69), and the activity of the granule-derived factors contributes to clearing invaders from the body (70). In the granular cells of the horseshoe crab, granules contain coagulation factors, precursors of various enzymes, and antibacterial molecules. Conversely, in the granular haemocytes of insects the granules mostly contain prophenoloxydase (proPO), the precursor of the enzyme responsible for encapsulation/melanization (71).

Phagocytic haemocytes are highly adherent cells that vary in number and phagocytic capacity between taxa. Filter feeding species usually have the most efficient phagocytes among invertebrates, while the phagocytic rates in crustacean phagocytic haemocytes are the lowest (72). *In vivo*, phagocytosis is facilitated by the presence of opsonins, plasma proteins that bind to the surface of microbes thereby enhancing recognition and uptake by phagocytes. Opsonins include soluble PRRs and cell adhesion proteins (in arthropods) (73), lectins, complement factors and other proteins in other taxa (20, 74).

During the response to an infection, a large proportion of the haemocytes involved in the reaction die (75, 76). The haemocyte levels are restored through different mechanisms.

- **Haematopoiesis** implies the differentiation of haemocytes from stem cells, a process that takes place only in insect embryos, whereas in larvae and adult insects the involved process is mitosis or differentiation (see below) (77–79).
- Another mechanism is **mitosis**, a phenomenon of self-renewal that allows some circulating haemocytes to expand in response to infections or other stressors (80, 81).
- Also, haemocyte levels can be restored by a process of **differentiation** from pro-haemocytes (rather than stem cells), as it occurs in insect organs in which quiescent pro-haemocytes, in response to the decreased levels of circulating haemocytes, differentiate into plasmatocytes or crystal cells (two types of mature granular cells) (82).
- **Heamocytosis** is the mechanism by which tissue-resident haemocytes migrate into the haemolymph, usually in response to an infection or another stressor (83).

These mechanisms, which ensure maintaining the adequate levels of immune effector cells, are classical repair and homeostatic mechanisms and are of great importance for ensuring the complete functionality of immune defense. Consequently, we need to consider them for a full understanding of immune memory at the level of the whole organism. The immune reactivity changes after the secondary challenge, both in acquired resistance and in recall responses, are paralleled by changes in haemocytes, in terms of global number and type of subpopulations. These changes are aimed at improving bacterial clearance through more efficient phagocytosis and enhanced stimulus-induced degranulation and consequent release of bactericidal factors. An interesting observation, although presently limited to *Drosophila* larvae, suggests that the defensive efficacy of haemocytes also depends on other mechanisms. A study leads to hypothesize that pathogens can modulate the haemocyte defensive capacity by interfering with hormonal levels/activity. Specifically, the parasites interfered with hormones regulating haemocyte membrane permeability, thereby inhibiting their differentiation and migration and consequently their ability to encapsulate the pathogen (for encapsulation, see chapter 5.2) (84).

**Figure 3:**
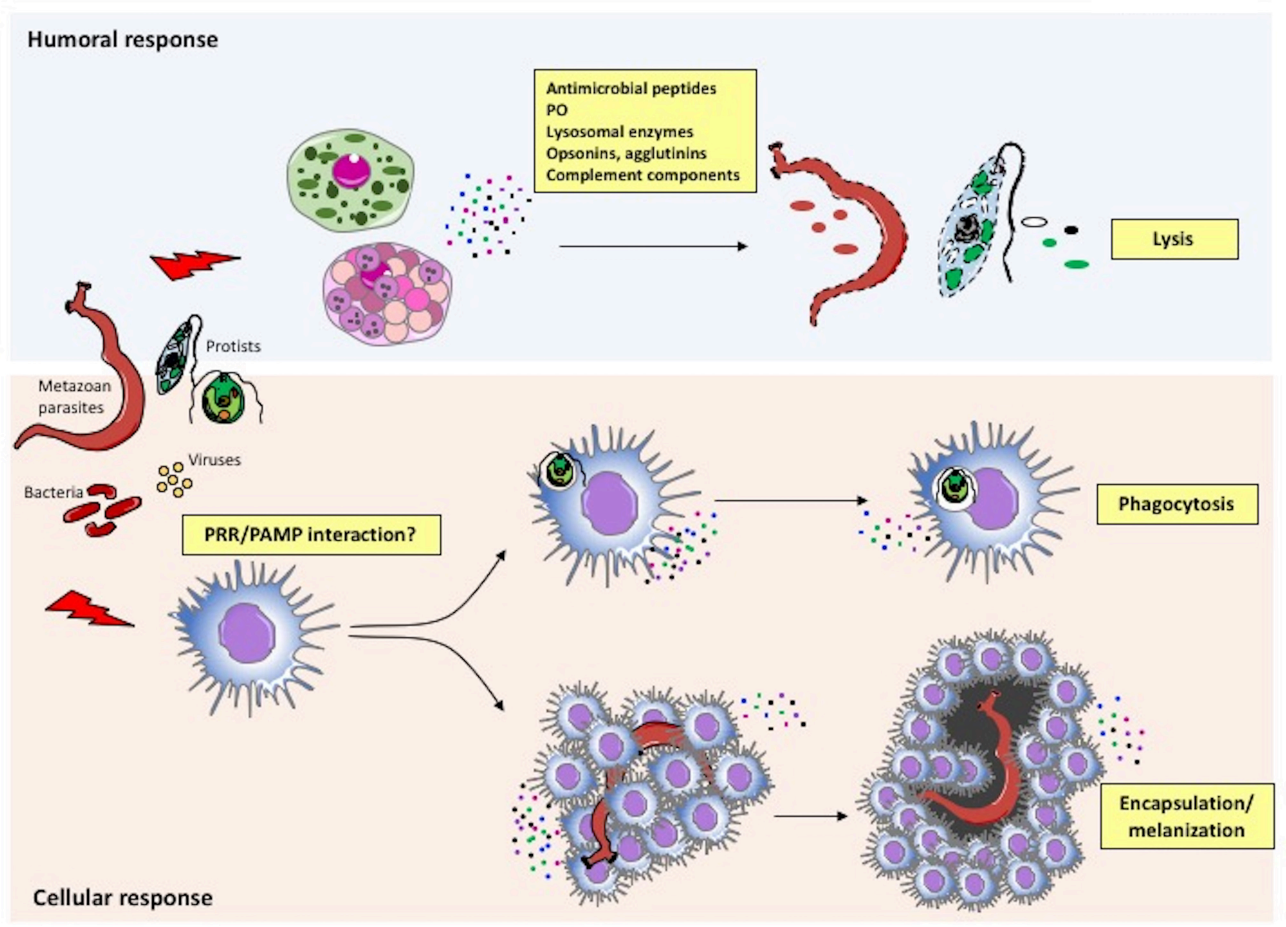
Immune defensive responses in invertebrates. Humoral and cellular effectors cooperate to achieve parasite/pathogen clearance. The immune system recognizes foreign agents (parasites, viruses, bacteria) and responds with the migration and production of immune cells (cellular response) and proteins (humoral response). More specifically, following recognition of pathogen-associated molecular patterns (PAMPs) or other molecules by PRRs on immune cells, circulating haemocytes within the haemolymph or immune cells in injured tissues neutralize the intruder by either phagocytosis or encapsulation/melanization. In parallel, the same or different immune cells release factors that are directly toxic for pathogens (antimicrobial peptides, agglutinins, etc.), or that improve or facilitate cell-mediated pathogen killing (PO, opsonins, complement components, etc.).

The main mechanisms of the invertebrate immune responses are summarized in Figure 3 and described below.

## Cellular immunity

### Phagocytosis and changes in circulating haemocytes

The core cellular defense function in invertebrates is phagocytosis, a process with two goals, defense from external invaders (by ingestion and elimination of intruding agents), and tissue homeostasis and remodeling (by clearing damaged cells). As an ancient trophic mechanism (85), phagocytosis was first observed in unicellular amoebae, soon after the divergence of plants. Phagocytosis became the specific defense function in the sentinel cells of social amoebae aggregates, cells that can be fully considered immune effectors, as they have developed Toll/Interleukin-1 receptor domain signaling pathways (86). Therefore, the ability to kill bacteria, either as a food source or for self-defense, emerged well before the appearance of metazoans. During metazoan development, it has been shown that phagocytosis of apoptotic bodies (the endogenous homeostatic function of phagocytes) primes cells to become reactive to injury and infectious signals. This priming allows cells to rapidly reach the wound site and to efficiently take up bacteria (87).

Several studies on invertebrate immune memory have addressed phagocytosis, both at the level of single phagocytic cells *in vitro* and as changes in the number of circulating phagocytes *in vivo*.

By measuring both the phagocytic index and the phagocytic rate, it is possible to distinguish the increase in phagocytosis due to an enhanced phagocytic ability of individual cells from the increase in the number of circulating phagocytes.

A study in the marine mollusc *Crassostrea gigas* investigated the cellular and molecular mechanisms of immune memory (54). Oysters primed *in vivo* with the inactivated pathogen *Vibrio splendidus* showed an enhanced response to a subsequent challenge with live *V. splendidus* in terms of increase of total haemocyte count, rapid regeneration of circulating haemocytes, phagocytosis, and expression of phagocytosis- and haematopoiesis-related genes. Since the phagocytic index was not different between the primed group and the unprimed controls, this suggests that the enhanced response of primed oysters was not due to priming-induced up-regulation of the effector functions of phagocytic cells, but it exclusively depended on an increase of the haemocyte number. The authors suggest that the secondary response is specific for *V. splendidus*, compared to other bacteria (*Vibrio anguillarum, Vibrio coralliilyticus, Yarrowia lipolytica, and Micrococcus luteus*). To support this hypothesis, however, the authors would need additional appropriate specificity controls and full time courses, and the use of bacteria strains that are phagocytosed to a similar extent in the unprimed phagocytic response. Thus, the data reported in this study allow us to identify a memory response in terms of increased number of phagocytes, without a reprogramming of phagocyte activity, while the evidence for specificity of recognition in the secondary response is not convincing.

Many studies in invertebrate immune memory aimed at demonstrating specificity, and used phagocytosis as parameter to evaluate the memory responses (57, 88–90).

The experimental design is however often conceptually unclear, encompassing several diverse phenomena under the definition of memory. In several studies, animals are primed *in vivo*, then haemocytes are collected and their phagocytic capacity against bacteria is measured *in vitro*. The functional assay used for assessing primed haemocyte memory (*in vitro* phagocytosis of bacteria) does not measure a memory response *sensu stricto* (the priming-induced re-programming of cellular responses measured upon a subsequent challenge), as it can likewise measure the unique sustained activation of *in vivo* primed/activated haemocytes.

As an example, in one of these studies the terrestrial arthropod *Porcellio scaber* was primed with three different inactivated bacteria (*Bacillus thuringiensis* strain 1 -Bt1-, Bt2, and *Escherichia coli*) (88). After two weeks, haemocytes collected from surviving animals were assessed for their ability to phagocytose Bt1, Bt2, and *E. coli*. Priming appeared to specifically increase phagocytosis, with higher uptake in the homologous combinations Bt1-Bt1 and Bt2-Bt2 compared to all heterologous combinations. The conclusion is that the organism has developed an immune activation that is specific for the priming bacteria. Although the result is clear, it is a pity that the authors do not specify if the differences in phagocytosis between homologous and heterologous combinations are due to differences in the number of phagocytes, or in their individual phagocytic ability. This would have provided information on the mechanism underlying this specificity. The ability of cells to discriminate between bacteria, i.e., memory at the cellular level, would rely only on the latter circumstance.

A similar study on the marine arthropod *Litopenaeus vannamei* showed that previous exposure of juvenile shrimps to inactivated pathogenic *Vibrio harveyi* caused an increased capacity of haemocytes to phagocytose this bacterium *in vitro*, while phagocytosis of Gram-positive *Bacillus subtilis* was unchanged (89). Conversely, shrimps primed with killed *B. subtilis* did not show an enhancement of phagocytosis toward the same bacterium. The authors suggested that *V. harveyi* can induce a specific memory. This study is in the same line as the one described above, i.e., it shows a specific phagocytic activity that may be due to sustained specific activation rather than to memory *sensu stricto*. Here the specific phagocytic activity was due to an increase in the number of phagocytic haemocytes rather than to the increased capacity of individual cells, showing memory at the organism level, rather than a memory response due to cellular reprogramming. An issue that, in any case, needs clarification is that, as in *C. gigas* (54), haemocytes from control shrimps were much more efficient in the uptake of *B. subtilis* compared to *V. harveyi*. Thus, an accurate determination of specificity in phagocytosis would need conditions in which the bacteria to be compared (in this case *B. subtilis* and *V. harveyi*) are taken up roughly at similar levels by unprimed haemocytes, thereby leaving enough room in the system to evaluate enhancements due to priming. This might not have been the case here with *B. subtilis*.

Another study investigated the development of immune memory in larvae of the terrestrial arthropod *Galleria mellonella* (57). Larvae were primed with the inactivated pathogens *Photorhabdus luminescens* and *B. thuringiensis*, and their haemocytes assessed at different times for number, phagocytic, and encapsulation activities. Larvae were then re-challenged with live bacteria and mortality was evaluated. The study shows that the infectious challenge was less severe, in term of mortality, if administered when the haemocyte activities were peaking. These results show that priming activates haemocytes and that resistance to subsequent infections depends on their activation status. Again, this does not directly demonstrate the induction of immune memory.

Following a similar experimental design, a study on larvae of the terrestrial arthropod *Bombyx mori* used phagocytosis for assessing the specificity of memory responses (90). Phagocytes from primed larvae were able to distinguish bacteria based on the Gram-type and could also discriminate between different strains of the same Gram-type. Indeed, both phagocytic index and rate increased (*in vivo* priming with inactivated bacteria + *in vitro* phagocytosis). Likewise, primed larvae that were challenged with live homologous bacteria (the same bacteria used for priming) showed the highest survival to the infection. This study describes a situation similar to the one mentioned above, i.e., the infectious challenge was administered when phagocytes were significantly active, after priming. This demonstrates specificity of immune activation but says little about the induction of immune memory.

As described above, invertebrate innate memory studies based on assessment of phagocytosis not always allow us to interpret the results unequivocally. In order to assess *bona fide* memory at the cellular level, a valid experimental design should consider both the rate and the index of phagocytosis, since the two parameters provide different information, and assess the number and percentage of the phagocytic cells within the haemocyte population. Also, phagocytosis of different bacteria and particles can vary significantly, and this should be taken into very careful account when evaluating the specificity of the memory effect.

Thus, based on the available information on the phagocytosis studies, we can say that invertebrates do develop immune memory, assessed as increased survival to infection in primed animals, survival that goes in parallel with an increase in both phagocytic index and rate in haemocytes (90). In several studies a priming-dependent long-lasting increase of phagocytic activity is evident, which is mainly due to the increase in the number circulating haemocytes rather than in their priming-induced re-programming, thereby showing a memory phenomenon at the organism level rather than induction of memory in immune cells. There are data in support of selectivity in bacterial recognition in memory responses (91–94), although more rigorous studies are needed to clarify the mechanisms underlying the phenomenon.

### Encapsulation

Encapsulation is a cell-mediated immune defense mechanism of invertebrates, which aims at clearing multicellular parasites, too large to be phagocytosed, from tissues and haemolymph. The haemocytes involved in this process adhere to each other and onto the surface of foreign particles through adhesion molecules, forming multilayer cellular sheaths (70). When the capsule is fully formed, haemocytes start a capsule melanization process, highly efficient in isolating the intruders (95). Melanization takes place upon the activation of proPO (96, 97), which is released by granular cells. Haemocytes also release cytotoxic factors for killing the invaders. The formation of capsules causes a marked reduction in the number of circulating haemocytes, which returns to normal levels in few days (76).

Like phagocytosis, encapsulation is a helpful parameter to evaluate immunocompetence in invertebrates, including evaluation of immune memory. In studies in *G. mellonella*, encapsulation of dextran beads was measured *in vitro* with haemocytes from animals primed with killed pathogens or lipopolysaccharide (57, 98). The haemocytes from primed animals were more active in encapsulating the foreign material, compared with cells from naïve animals. Although run *in vitro*, this phenomenon can be defined as priming-induced sustained response, one of the types of memory-like responses (see paragraph Haemocytes).

In a pioneering study on immunological memory in the urochordate *C. intestinalis*, a first injection of human or duck erythrocytes in the tunic elicited a primary defensive reaction mainly based on phagocytosis, while after challenge with the same erythrocytes the reaction shifted to encapsulation (56). This shift involves the activation of morula cells, cells mostly residing in the tunica, which are the effector cells that build the capsule around foreign objects (99). We can consider the shift from phagocytosis to encapsulation as a *bona fide* memory response, in which the response to challenge is more efficient than the first one in ensuring isolation of the foreign material and in accelerating its clearance. Interestingly, encapsulation exceeds phagocytosis in sexually mature animals, suggesting a more efficient defensive system.

## Humoral immunity

In invertebrates, the effectors of humoral immunity are soluble factors secreted by granulocytes, such as lectins, agglutinins, antimicrobial peptides (AMPs), complement-like factors, and proPO, the precursor of the enzyme phenoloxidase (PO; Figure 3). These factors act in concert with phagocytes to fight microorganisms and other foreign agents that have entered the body by overcoming physical and chemical barriers (100). Lectins are soluble and membrane-associated molecules capable of pathogen recognition, involved in defensive mechanisms such as agglutination, complement-mediated opsonization and lysis (101). Agglutinins are antibody-like non-immunoglobulin molecules present in the haemolymph that, together with lectins, form the pathogen-recognizing lectin-agglutinin system (102). AMPs are small soluble peptides with direct toxic activity. ProPO and complement are cascade systems, present as inactive precursor molecules in steady state. Hereafter, we will describe the possible involvement of proPO, complement, and AMPs in immune memory.

### Prophenoloxidase

The PO precursor proPO is constitutively synthesized by a subset of haemocytes, the granular cells, and released and activated in response to microbial compounds or endogenous factors produced upon tissue damage (96). Active PO leads to melanization of microorganisms or damaged tissues. Melanin acts as a physical shield that prevents or delays parasite growth. PO initiates melanin biosynthesis by oxidizing monophenols and diphenols to orthoquinones, which then polymerize into melanin. The toxic quinone intermediates also contribute to the defensive reaction (95). As melanization is one of the major innate defense responses in invertebrates, it is important to evaluate PO activity in studies on immune memory.

Only one study, in larvae of *B. mori*, has addressed PO activity in immune memory (90). The PO enzymatic activity in larval haemolymph was measured 3 days after priming, corresponding to the highest phagocytic rate (measured as number of phagocytosing cells over the total number of haemocytes). However, PO activity did not positively correlate with survival of primed larvae to a second infection. It should be stressed that PO is involved in melanization, and that melanization only starts when phagocytosis is not sufficient for eliminating the infectious agents, which may not be the case here. In any case, since the experimental design did not formally address a memory phenomenon, this provides no information on the role of PO in memory responses.

### Complement

The complement system is one of the major defensive tools of all metazoans. A genomic-evolutionary approach allowed researchers to identify several complement components even in *Cnidaria*, a very ancient phylum (103). Most of the information on the invertebrate complement components is based on genomic sequences, while identification of proteins and evaluation of their biological role are limited to few models, such as sea urchin (104–107) and tunicates (60, 61, 108, 109). Taking into account genomic and functional data, evolutionary scientists conceived a primitive version of the complement system with C3 as the central component. Upon proteolytic cleavage, C3 generates the opsonizing factor C3b and the chemotactic anaphylatoxin C3a. The identification of factor B (Bf) and mannose-binding protein-associated serine proteases (MASPs), responsible for C3 activation, suggests the existence of two complement activation cascades similar to the mammalian alternative and lectin pathways. C6 homologs have been identified, as well as other genes belonging to complement cascade, but their function is still unknown (103).

To date, only one study has addressed the involvement of three complement genes (C3, C6, Bf) in immune memory, specifically in a recall response (53). In this study, the cephalochordate *Branchiostoma belcheri* was primed with inactivated bacteria (*V. anguillarum, E. coli*, or *Staphylococcus aureus*) and later re-exposed to the same bacteria. After challenge, the expression of the alternative complement components Bf, C3, and C6 was significantly higher and peaked earlier compared with the first exposure. The authors show specificity in the induction of the memory response, as priming with one bacterium enhances the secondary response to the same bacterium. Interestingly, animals primed with *E. coli* respond better to *V. anguillarum* than unprimed animals, but the reverse is not true (although the authors claim otherwise). Priming with *S. aureus* does not affect the response to *V. anguillarum* and vice-versa. Thus, the authors' claim that memory is class-specific (Gram-negative vs. Gram-positive bacteria) is only based on the non-reciprocal capacity of *E. coli* (Gram-negative) to induce memory to a *V. anguillarum* challenge, and therefore it needs additional proof. The study did not include a functional validation of the up-regulation of complement gene expression (e.g., complement-dependent bacterial clearance), thus the relevance of this memory response in term of improved defensive functions is unknown.

### Antimicrobial peptides

Antimicrobial peptides/proteins are of particular importance for invertebrate defense, as they are toxic for bacteria, yeasts, filamentous fungi, protozoa, and enveloped viruses, thereby preventing infections (100, 110, 111). Despite a large structural diversity, AMPs have in common the ability to permeabilizing microbial membranes, leading to cell death. In general, e.g., in marine annelid, shrimp, oyster and horseshoe crab, AMPs are constitutively expressed, stored in circulating granular haemocytes, and secreted during an acute immune response (112–115), while in *Drosophila* anti-fungal peptides are induced upon phagocytosis (116, 117). AMPs take part in determining the composition of the microbiota associated with the host (118), as they are robustly present in tissues/organs that are highly colonized by bacteria and in those that face external environments (terrestrial and aquatic). Thus, studies on immune memory have also considered AMPs as possible effectors of the memory responses.

Several studies on memory have examined the antimicrobial activity of cell-free haemolymph, which is the way of testing the presence of AMPs. These studies observed an increase in antibacterial activity (cell growth inhibition) in the haemolymph of primed animals (57, 98). Larvae of *B. mori* primed with inactivated Gram-negative or Gram-positive pathogens had significantly higher haemolymph antibacterial activity than unprimed control larvae, with the highest activity observed upon challenge with the same microorganism used for priming (90). These results confirm the establishment of a priming-induced memory, which is evident in the form of a typical recall response, and support the hypothesis that some degree of specificity is indeed present in invertebrate memory responses.

## Host-parasite models

Possibly, the best way of examining immune memory in invertebrates is the use of infection models in which animals recovering from a first infection are re-infected with the same or with a different pathogen. Indeed, in invertebrates it is possible to set up *in vivo* experiments and use naturally occurring pathogens, thereby obtaining realistic models and reliable results. However, the short life span of the animals can represent a significant drawback.

One of the models used is that of *Anopheles* and malaria parasites, particularly important for its impact on human health, because plasmodia are transmitted to humans by female *Anopheles* mosquitoes. The successful development of the malaria parasite in the mosquito depends on several environmental factors and on the lifespan of the mosquito, which should be long enough to allow the parasite to complete its cycle within the host [a circumstance occurring only in 10% of female *Anopheles gambiae*; (119)]. This biological constraint makes the mosquito-plasmodium model not fully suitable to study immune memory, because primed mosquitoes would likely die before the primary infection is resolved. In a study on immune memory in this model, the authors were forced to challenge the mosquitoes with plasmodia before the animals had resolved the first infection (120). The study shows that the primary infection causes a life-long rise in the number of granular cells, due to the activation of a differentiation process by a lipoproteic signal in the haemolymph, induced by the infection (121). Since the animals never clear the infection, the presence of the differentiation factor and the consequent presence of granular cells are expected. The authors hypothesize an increased resistance to plasmodia after re-infection, based on the number of new oocysts in the mosquito midgut, which is lower than that of the oocysts (still present in the midgut) coming from the first infection, and correlate it to the lipoprotein-induced increase in granular cells. The authors propose a role for the gut microbiota in reducing plasmodial infection, and hypothesize that that the gut damage produced by plasmodial infection allows for the development of immunity against gut microbiota, thereby reducing the microbiota-dependent anti-plasmodial effect.

That this is a mechanism of memory *sensu stricto* is difficult to say, as the continuous presence of the parasites (as the first infection was not resolved before re-infection) leads to hypothesize a complex reaction to multiple challenges, without extinction of response between challenges, and also encompassing a possible competition of older parasites with the new infection. Another finding that casts some doubts on the possibility that the final result is a memory response is that no evidence of encapsulation is shown after the secondary challenge. In fact, it is well known that encapsulation is the most effective mechanism of anti-plasmodium defense in *Anopheles*, and we would expect that, similarly to *C. intestinalis* (56), a memory response resulting in better protection would imply a shift from a primary reaction (lysis brought about by granular cells in the case of *An. gambiae*) to encapsulation.

A similar problem is present in immune memory studies on the freshwater mollusc *B. glabrata* infected with its parasite, the trematode *Schistosoma mansoni* (122). Also in this case, the infectious challenge was administered to the snail before the first infection had resolved, resulting in an overlapping re-infection. These experimental conditions allowed for an interesting study on immune responses to multiple overlapping infectious challenges, although not fully suited for assessing memory responses. The study reports a number of genes up-regulated after re-infection and a shift from cellular (encapsulation) to humoral immune response (lysis) (122). Among the up-regulated genes, the authors identified those for the fibrinogen-related proteins FREPs as possible immune effectors (123–126). However, this does not seem to be the case, as knocking down FREP genes did not significantly affect the course of the infection. The other, very interesting result is the shift from cellular to humoral immune responses upon re-infection. This was proposed based on the observation that both infected/re-infected animals and animals that received only the second infection plus the cell-free haemolymph of infected snails had low number of parasites and no encapsulation was evident. The soluble factor conferring resistance was not identified, and it is not even clear if it is produced by the host or by the parasite. In the absence of a number of controls necessary to clarify the many outstanding questions, and with the notion that encapsulation is a very efficient defense mechanism (better than lysis), it appears that this study is addressing the immune responses to re-infection/multiple overlapping infections, but it is not really informative on mechanisms of immune memory.

## Epigenetics

The ability of vertebrate innate immune cells to mount a different transcriptional response, when challenged with pathogens or other stressors, is due to an extensive epigenetic re-programming, leading to changes in gene expression and cell physiology (127). The epigenetic changes act at the level of chromatin by regulating its accessibility to the transcriptional machinery of the cell. Before transcription, this can be achieved by the modifications of chromatin, such as methylation of DNA or histones, or acetylation and deacetylation of histones. Post-transcriptionally, gene regulation has been attributed to microRNAs (miRNAs), which can regulate mRNAs either by inhibiting translation or by promoting its degradation (128).

The most complete studies on the epigenetic re-programming at the basis of immune memory took advantage of the peculiar characteristics of the planarians. Planarians, which are acoelomate invertebrates, possess a pool of adult pluripotent stem cells, termed neoblasts. These freely moving cells, present in the parenchyma, are the only dividing cells during normal postembryonic development and regeneration (129, 130). Neoblasts could also differentiate into reticular cells, mesenchymal migrating cells with immune functions, which are the planarian equivalent of circulating phagocytic cells (131, 132). A study in the freshwater platyhelminthe *Schmidtea mediterranea* demonstrated that *S. aureus*-primed animals (fed with live bacteria) could clear a subsequent bacterial challenge much faster than unprimed animals, a typical recall response (52). The finding is that *S. aureus* (but not *Legionella pneumophila* or *Mycobacterium avium*) induced the expression of Smed-PGRP-2 (a peptidoglycan receptor, likely activated by binding with *S. aureus*), which triggered the expression of the Smed-setd8-1 histone methyltransferase gene, and this in turn up-regulated the expression of the *Smed-p38 MAPK* and *Smed-morn2* genes, allegedly involved in the downstream steps of the anti-bacterial responses. The second infection with *S. aureus* induced the expression of *Smed-p38 MAPK* and *Smed-morn2* much earlier than upon the first infection. An increased level of lysine methylation was detected in neoblasts from primed worms compared to naïve, and correlated with expression of the Smed-setd8-1 histone methyltransferase gene, as demonstrated by knockdown experiments (55). The involvement of neoblasts in immune memory was demonstrated by RNAi silencing of Smed-H2B, a neoblast-specific histone necessary for cell survival, in primed animals. Remarkably, the kinetics of bacterial clearance in neoblast-depleted re-infected animals was indistinguishable from the response to the first infection, thus specifically indicating a loss of immune memory. As further proof of neoblast-dependent memory, tissues from primed donors, with or without neoblasts (ablated by irradiation) (133), were transplanted in naïve animals that were then infected with *S. aureus*. While grafting of intact tissues from primed donors induced enhanced bacterial clearance, as in a memory response, the neoblast-depleted tissue transfer did not have any enhancing effect.

As the results described above are convincingly showing the involvement of neoblasts in memory responses, a puzzling observation is that neoblast depletion before priming did not affect the development of memory. This might lead to the hypothesis that the infection with *S. aureus* (but not with *L. pneumophila* or *M. avium*) would cause a significant loss of reticular cells (dying while combating the pathogen) and a consequent powerful induction of neoblast proliferation and differentiation, to compensate the loss. As the kinetics of neoblast proliferation has not been assessed in this study, we do not know whether the second infection takes place during the peak of neoblast hyperproliferation (above steady-state levels) or after steady-state has been re-established. In the former case, the observed memory phenotype, including all the increased levels of immune-related genes, could be attributed to the presence of a higher number of neoblasts rather than to quantitative changes in individual cells.

In any case, in this scenario the Smed-set8-1 methyltransferase emerges as an important epigenetic signature involved in immune memory, as the gene is not expressed when animals are infected by bacterial species (*L. pneumophila* and *M. avium*) that do not induce memory, and, consequently, no Lys methylation occurs.

As a final comment, it is interesting to note that the two bacterial strains that do not seem to induce memory are not natural pathogens for planarians, and the infection was artificially induced in the lab. The course of such artificial infections is most likely not the same as that of the natural pathogen *S. aureus*, and therefore the lack of memory induction might simply be due to differences in the infection course/profile.

### Transgenerational immune priming

Epigenetic re-programming has been invoked in transgenerational immune priming (TgIP), i.e., the transfer of immune memory from a primed parent to the offspring and the following generations. By causing transmissible changes in gene expression profiles (134), this transfer of immune memory allows the offspring to better survive to pathogens populating the same environment of the parents (135). However, it should be noted that this phenomenon might also influence other complex parameters, such as fecundity and longevity. For example, in the moth *Manduca sexta* TgIP is beneficial for the survival and growth of the offspring larvae, but it impairs reproduction of the adult offspring (136).

Epigenetic re-programming is at the basis of TgIP. The role of histone acetylation in the transcriptional re-programming associated with transgenerational immune priming was hypothesized in *G. mellonella* larvae fed with the pathogen *Serratia entomophila*. The pathogen tipped the balance between HDAC (histone deacetylases) and HATs (histone acetyltransferases) expression in favor of HDAC both in the midgut of infected larvae and eggs, compared to non-infected controls (137, 138). In the same *G. mellonella* model, the availability of transcriptomic data has made possible to identify miRNAs that are differentially regulated during infection with a parasitic fungus (*Metarhizium anisopliae)* or with pathogenic bacteria (*S. entomophila)*. For example, infection with *S. entomophila* induced an abundant presence of api-miR-263a in eggs, a finding that suggests the possibility that infection-induced changes in miRNA levels in eggs could take part into the transgenerational transfer of immune memory. Conversely, dps-miR-200b was silenced during infection with *M. anisopliae*, and its target genes were consequently upregulated (139).

A study on the crustacean *Artemia franciscana* focused on transfer of immune memory from animals primed with *Vibrio spp*. to the progeny. Immune memory in primed parent animals and in their unprimed progeny was measured in terms of increased survival upon exposure to *Vibrio*. The transcriptomic data indicate a correlation between animal resistance and up-regulation of some immune-related genes, such as heat shock protein 70 (HSP70), high mobility group box 1 (hmgb1) and peroxinectin (140). Conversely, the transcription level of other immune-related genes, i.e., Dscam and lipopolysaccharide- and beta-1,3-glucan-binding protein (lgbp), did not change (140). It should be underlined that the functional role of the proteins encoded by these genes has not yet been identified in *Artemia*. The levels of acetylation and methylation of histones H3, H4, and H3K4me3 showed a random pattern throughout the following generations, suggesting that the epigenetic mechanisms based on histone acetylation and methylation do not seem to contribute to the up-regulation of immune-related genes in transgenerational immune priming in *Artemia* (140). Very interestingly, the situation is different when TgIP is induced by an abiotic stress. Heat shock could induce immune memory (increased production of HSP70, tolerance to lethal heat shock, and resistance to pathogenic *V. campbellii*), and these traits can be transmitted to three successive, unexposed generations (18). At variance with *Vibrio*-induced TgIP, the TgIP induced by heat shock was clearly associated with epigenetic changes, and in particular with acetylation of histones H3 and H4. Taken together, the results of the two studies suggest that TgIP in *Artemia* is based on different mechanisms, depending on the type of priming agent/event, with histone modifications probably involved in TgIP due to abiotic stress but not in that induced by *Vibrio* infection.

## Conclusions

Since invertebrates are a very heterogeneous group of animals, the study of innate immune memory has been focused mainly on species attracting interest for their commercial, ecological, epidemiological, or evolutionary importance. The majority of studies on immune memory are phenomenological studies of whole organisms, whereas the molecular basis of memory is still poorly explored.

The main endpoints of immune activation, the same used for assessing immune memory, are phagocytosis and encapsulation of intruders (clearance), which are functions pertaining to phagocytic haemocytes, the counterpart of vertebrate macrophages, and/or granular haemocytes, which are loosely similar to vertebrate polymorphonuclear leukocytes. These cells, either directly or in concert with humoral factors, are responsible for the improved capacity of clearing infectious agents and the consequent increased survival.

In the few models in which memory was formally demonstrated, the immune potentiation at challenge depends on a higher, quicker and cell type-specific production of the haemocytes responsible for phagocytosis (*Crassostrea*) and encapsulation (*Ciona*). This central event can be observed also in mice, in which establishment of protective memory in response to a priming with β-glucan is paralleled by the expansion of myeloid precursors in the bone marrow (141).

In invertebrates, the variations in specific haemocyte subpopulations depend on several mechanisms (differentiation, haematopoiesis, mitosis), which are differentially active depending on developmental (sexually immature vs. sexually mature) and physiological biases (larval stages vs. adults, stem vs. differentiated cells).

**Figure 4:**
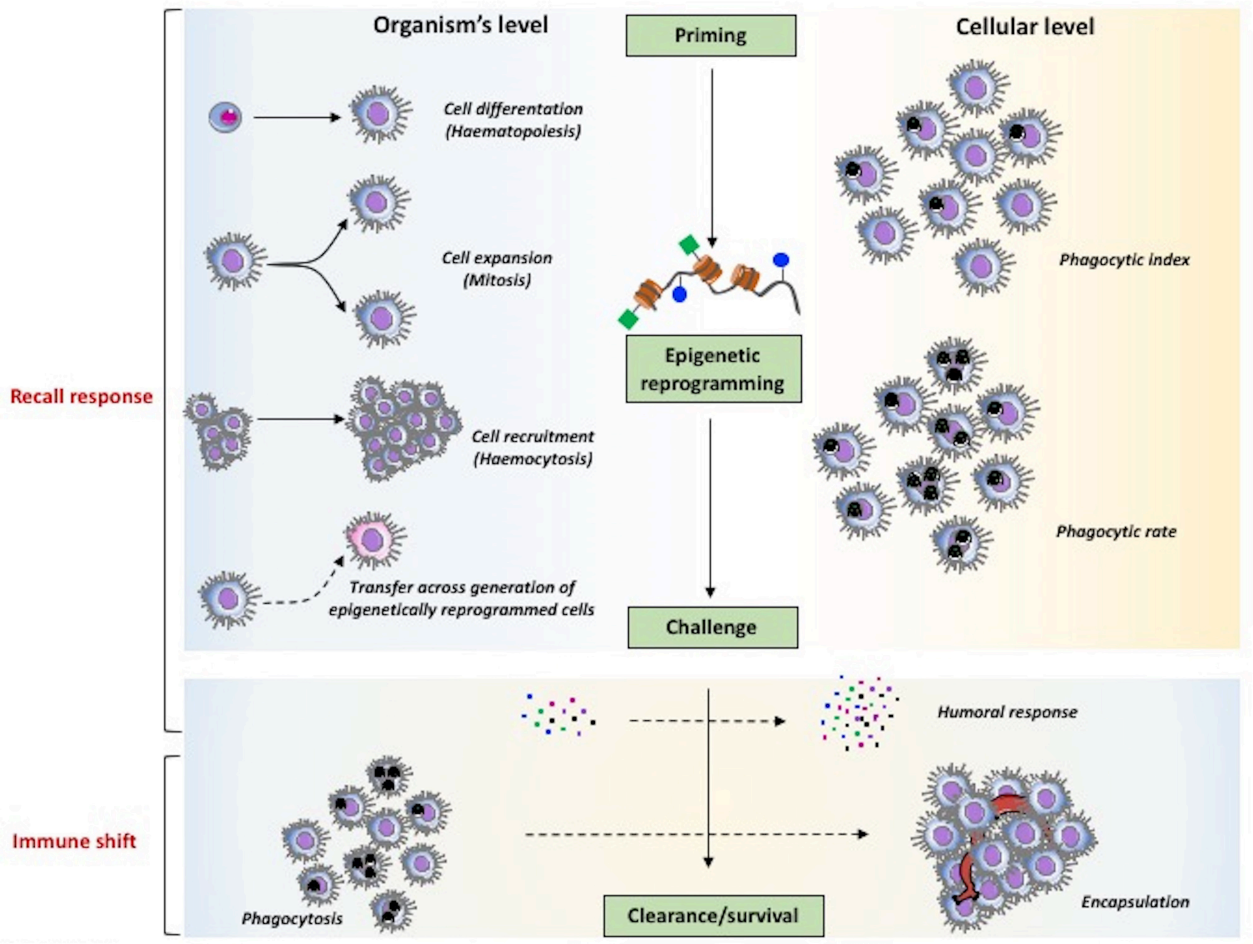
The major mechanisms of innate memory in invertebrates. The two major phenomena underlying the capacity of invertebrates to mount a more effective defensive response after priming, speaking *sensu stricto*, are the recall response (upper part) and the immune shift (lower part). As general mechanism (center part), priming is expected to induce epigenetic reprogramming that, upon challenge, determines improved clearance of parasites and enhanced survival. At the level of the whole organism (left part), memory can encompass mechanisms leading to an increase in the number of immune cells at the site or reaction (haematopoiesis, mitosis, haemocytosis), and also the capacity of transferring resistance across generations. At the cellular level (right part) it is also possible to observe increased effector functions in individual cells (e.g., an increased phagocytic rate vs. phagocytic index). Mechanisms that are observed both at the global and cellular levels (lower part) encompass the increased production of soluble immune mediators and the shift of immune response from an initial protective reaction (e.g., phagocytosis) to a more effective mechanism (encapsulation).

As described earlier, in planarians the cells involved in memory are the stem-like neoblasts, which are re-programmed epigenetically in order to achieve enhanced resistance to reinfection with *S. aureus*. This finding may suggest that also in coelomate invertebrates the epigenetic re-programming could be the molecular mechanism at the basis of haemocyte expansion (Figure 4). It is important to underline the finding that epigenetic signatures and the associated immune memory can be transmitted across generations (TgIP), although the process at the basis of this transmission is still unknown (Figure 4). It is interesting to see that in honeybees priming of mothers increases resistance to infection in the unexposed progeny and that this TgIP correlates with an increased number of differentiated haemocytes (142).

In vertebrates, the epigenetic re-programming has been invoked as mechanism at the basis of innate memory in monocytes (7), whereas nothing is known on epigenetic modulation of stem cells or heritability of innate memory.

Thus, by taking in consideration only the limited number of studies that address the memory phenomenon in its strictest meaning, we can identify three main characteristics of immune memory in invertebrates:

- expansion/differentiation/recruitment of haemocytes is generally at the basis of immune memory;
- immune memory can be transmitted to the progeny;
- epigenetic re-programming in many cases underlies memory and its transgenerational effects.

This does not take into account a wealth of other important studies, in which the memory phenomenon has been addressed from a wider point of view. In these studies, many other factors have been identified that can contribute to the improved defensive performance, in addition to those mentioned above. For this, we refer the reader to some exhaustive reviews (10, 14, 15, 62).

Overall, the evidence reviewed here shows that invertebrates, devoid of adaptive immunity, can generate protective long-term immune memory, likely based, from a molecular point of view, on epigenetic re-programming at the level of stem cells. The fact that immune memory can be transmitted to the following generations is an important finding, which has not been yet described in vertebrates. The only indirect evidence that innate memory could be transmitted across generations is that transgenerational epigenetic effects and transgenerational epigenetic inheritance are well-known phenomena in vertebrates including humans (143).

Understanding invertebrate immune memory in terms of induction, establishment, and even heritability will help us in better understanding the evolution and differentiation of immune mechanisms.

## Author contributions

DM and RM studied the literature and drafted the manuscript. PI and DB critically revised and finalized the manuscript. PI conceived and prepared the figures.

### Conflict of interest statement

The authors declare that the research was conducted in the absence of any commercial or financial relationships that could be construed as a potential conflict of interest.
